# A single mutation in the *cis*-acting replication element identified within the EV-A71 2C-coding region causes defects in virus production in cell culture

**DOI:** 10.1080/22221751.2021.1977590

**Published:** 2021-10-17

**Authors:** Juemin Xi, Chunxia Ma, Zhizhong Wei, Bin Yin, Siwen Zhao, Wenqi Quan, Jing Yang, Jiangang Yuan, Boqin Qiang, Fei Ye, Xiaozhong Peng

**Affiliations:** a Institute of Medical Biology, Chinese Academy of Medical Sciences, and Peking Union Medical College, Kunming, People’s Republic of China; b The State Key Laboratory of Medical Molecular Biology, Department of Molecular Biology and Biochemistry, Institute of Basic Medical Sciences, Chinese Academy of Medical Sciences and Peking Union Medical College, Beijing, People’s Republic of China; c NHC Key Laboratory of Biosafety, National Institute for Viral Disease Control & Prevention, Chinese Center for Disease Control and Prevention, China CDC, Beijing, People’s Republic of China

**Keywords:** Enterovirus A71, cre, 2C-coding region, virus replication, cellular factors

## Abstract

Enterovirus A71 (EV-A71) can cause hand, foot and mouth disease with neurological and systemic complications, most frequently affecting children and infants. We describe a *cis-*acting replication element (*cre*) with a conserved stem-loop structure within the EV-A71 2C-coding region. By site-directed mutagenesis and reverse genetics using the EV-A71 full-length genome and the EV-A71 replicon containing the firefly luciferase reporter gene in place of the P1 region, the stem-loop structure and the AAACA in the loop of the *cre* were confirmed to be required for the EV-A71 replication phenotype. EV-A71 genomes containing a mutation at the first or third A residue of AAACA could not be recovered. Insertion of a wild-type *cre* from EV-A71 or poliovirus in the 5’UTR led to successful recovery of the replication of nonviable mutants. Furthermore, the *cre* mutants showed lower binding capacity with the host cellular factor IGF2BP2, knockdown of which resulted in a significant decrease in EV-A71 production. All the available evidence shows the location independence but functional importance of the interaction of the *cre* with the cellular host for efficient production of EV-A71, contributing to the growing body of knowledge regarding picornavirus *cre*s.

## Introduction

Enterovirus A71 (EV-A71) is one of the most common pathogens causing hand, foot and mouth disease (HFMD) in children and infants especially in Asia-Pacific region [1–3]. A proportion of the patients develop neurological manifestations and even die [4–8]. EV-A71 belongs tothe species *Enterovirus A* of the genus *Enterovirus* of the family *Picornaviridae*. EV-A71 strains are classified into the genotypes A, B, C, D [9, 10] and into the recently proposed genotypes E, F and G, which were mostly identified in the Central African Republic, Madagascar and India, respectively [10–13]. Genotypes B and C individually consist of subgenogroups B0-B5 and C1-C5 [14]. The EV-A71 genome is a single-stranded, positive-sense RNA that is approximately 7.4 kb in length and contains an open reading frame encoding a polyprotein, which is proteolyzed into the precursor proteins P1, P2 and P3. The P1 precursor protein is further processed into four structural proteins, namely, VP1, VP2, VP3 and VP4. The P2 and P3 precursor proteins are proteolyzed into the nonstructural proteins 2A, 2B, and 2C and 3A, 3B, 3C, and 3D, respectively [15]. The low-molecular-weight viral protein genome-linked (VPg) covalently binds to the 5’ untranslated region (UTR) [16].

In the life cycle of positive-strand RNA viruses, the central process is RNA-templated RNA synthesis, which can be initiated by two primary mechanisms: *de novo* initiation [17, 18] and primer-dependent initiation [19, 20]. As in the case of picornaviruses, the viral RNA-dependent RNA polymerase links the 5’-terminal U (UMP) to the hydroxyl group of tyrosine in virus-encoded VPg using a *cis*-acting replication element (*cre*) as a template during protein-primed initiation. The *cre* is within a small RNA hairpin that contains a conserved ^1^GXXXAAAXXXXXXA^14^ sequence in the loop [21–23]. Within various picornaviral genomes, hairpin structures are likely to be present but located in different regions, although they share several common features. A study on foot-and-mouth disease virus (FMDV) discovered a *cre* in the 5’UTR of the genome adjacent to the internal ribosome entry site [24]. The first indication of a *cre* in the coding sequences of a picornavirus came from the finding that deletion of the P1 coding region of human rhinovirus type 14 (HRV14) resulted in the inability of the replicons to replicate [25]. Similar elements were later found in the VP2-coding sequences of Theiler’s virus and Mengo virus [26]. *Cre* sequences have also been identified within the 2C-coding regions for both poliovirus (PV) [27, 28] and coxsackievirus B3 [29], the 2A^pro^-coding sequence of HRV2 [30], the 3D^pol^-coding sequence of hepatoviruses [31], and the 3’UTR of severe acute respiratory syndrome coronavirus 2 (SARS-CoV-2) [32] which belongs to the lineage B Betacoronavirus family. In genetic and biochemical studies of both PV and HRV2, substitutions mapping to the first two consecutive A residues of AAACA in the loop of the *cre* severely reduced RNA infectivity and the *cre* RNA’s ability to serve as a template for VPg uridylation [28, 30]. In PV, replication competence remained when the *cre* was moved from the 2C protein-coding region to the 5’UTR [23]. To date, however, studies for such an element in the EV-A71 genome have not been productive.

Here, we described a *cre* with a conserved stem-loop structure within EV-A71 2C-coding region and confirmed the location-independent but functionally important role of *cre* in EV-A71 replication using a series of mutants derived from either a replicon or a full-length genome by mutagenesis and reverse molecular genetics. The first and third A residues of the AAACA in the loop of the *cre* were of critical importance for the replication phenotype. A wild-type *cre* from EV-A71 or PV inserted into the 5’UTR of the EV-A71 genome could rescue viruses. Furthermore, we found that the *cre* mutants had lower binding capacities with the cellular factor insulin-like growth factor 2 mRNA-binding protein 2 (IGF2BP2). Knockdown of IGF2BP2 expression led to a significant decrease in EV-A71 production. These results indicate that the *cre* is crucial for the efficient replication of EV-A71 and reveal that the *cre* functions together with the cellular host during EV-A71 infection.

## Methods

### Cell lines and virus

Vero cells and KMB17 cells obtained from the Institute of Medical Biology, Chinese Academy of Medical Sciences, and Peking Union Medical College, China, were maintained in minimum essential medium (MEM) (HyClone) supplemented with 10% fetal bovine serum (FBS) (Gibco) and 1% penicillin/streptomycin at 37°C with 5% CO_2_. 293 T cells were cultured in Dulbecco’s modified Eagle’s medium (DMEM) with 10% FBS and 1% penicillin/streptomycin at 37°C with 5% CO_2_. T98G cells were cultured in modified MEM supplemented with 10% FBS, 1 mM sodium pyruvate, 1×MEM nonessential amino acid solution (Thermo Fisher Scientific) and 1% penicillin/streptomycin at 37°C with 5% CO_2_. Human EV-A71 strain 87-2008 Xi’an Shaanxi (GenBank ID: HM003207.1) was kindly supplied by Professor Wenbo Xu in Chinese Centre for Disease Control and Prevention. The EV-A71 infection was performed in Vero cells, KMB17 cells and T98G cells. The titers of EV-A71 and its mutant derivatives were determined by plaque assay in Vero monolayers.

### Plasmid construction

Viral RNA was extracted by using a QIAamp® Viral RNA Mini Kit (Qiagen) following the manufacturer’s instructions. Viral cDNA was synthesized from genomic RNA using the SuperScript^™^ III First-Strand Synthesis System (Thermo Fisher Scientific) with oligo-dT primers. To construct the full-length cDNA clone of EV-A71, the pBR322 vector and the restriction digestion sites SalI and BspEI were used. A T7 RNA polymerase promoter (T7) and a poly (A) tail were engineered at the 5’ and 3’ ends of the full-length viral cDNA for *in vitro* transcription and for generation of the authentic 3’ end of the RNA transcript, respectively. The whole genome of EV-A71 was amplified by a primer pair (forward primer: ACGCGTCGACTTAATACGACTCACTATAGGG TTTAAAACAGCCTGTGGGTTG, reverse primer: GGCTCCGGATTTTTTTTTTTTTTTTTTTTTTTTTGCTATTCTGGTTATAACAAATTTACCCCCA) using Invitrogen^™^ Platinum^™^ SuperFi^™^ PCR Master Mix (Thermo Fisher Scientific) in a 50 μl PCR volume, containing 25 μl of 2× Platinum^™^ SuperFi^™^ PCR Master Mix, 2 μl of 10 µM forward primer, 2 μl of 10 µM reverse primer, 10 μl of 5× SuperFi^™^ GC Enhancer, 1 μl of cDNA and 10 μl of nuclease-free water. PCR was performed with the following parameters: initial heating at 98°C for 30 s, followed by 35 cycles of 98°C for 30 s, 60°C for 10 s, and 72°C for 3 min 40 s, and a final elongation at 72°C for 5 min. The plasmid containing the full-length cDNA of EV-A71 (pBR322-EV-A71) was analyzed by restriction digestion and confirmed by sequencing.

To construct different EV-A71 *cre* clones, eight pairs of primers were designed for site-directed mutagenesis (Table S1), which was performed with a QuikChange Lightning Site-Directed Mutagenesis Kit (Agilent Technologies). One microliter of plasmid (< 100 ng), 5 μl of 10× QuikChange Lightning Buffer, 1 μl of dNTP mix, 1 μl of 10 µM forward primer, 1 μl of 10 µM reverse primer, 1.5 μl of QuikSolution reagent, 1 μl of QuikChange Lightning Enzyme and 38.5 μl of ddH_2_O were used in a 50 μl reaction system. PCR was performed as follows: initial heating at 95°C for 2 min, followed by 18 cycles of 95°C for 20 s, 60°C for 10 s, and 68°C for 3 min 40 s, and a final elongation at 68°C for 5 min.

To construct dual-*cre* clones, the mutations A113 T and G117 T were introduced into pBR322-EV-A71 to produce the NsiI site. The plasmid was then restriction digested by NsiI and ligated with the annealed *cre* fragments with NsiI sites using T4 ligase (NEB) at 16°C for 4 h. The bacterial strains XL10-Gold and JM110 were used as the *Escherichia coli* hosts for the construction and propagation of the subclones and the full-length cDNA clone. The final plasmids were confirmed by DNA sequencing.

### Replicon construction

The pBR322-EV-A71 plasmid was further used to generate the firefly luciferase EV-A71 replicon (EV-A71-Fluc). A713 T and C717G for XbaI and T3298C for AgeI were produced by site-directed mutagenesis in the 5’UTR and P1 region of the EV-A71 genome, respectively. The P1 region was then deleted by XbaI and AgeI restriction digestion and replaced by the firefly luciferase reporter gene.

### In vitro RNA transcription and transfection

The plasmid pBR322-EV-A71 and its mutants were amplified in bacterial strain JM110 and purified using the PureLink® HiPure Plasmid Filter Midiprep Kit (Thermo Fisher Scientific). Then, 2.5 μg of plasmid was linearized with the restriction enzyme BspEI in a 50 μl reaction at 37°C for 2 h. The T7 RiboMAX™ Large Scale RNA Production System (Promega) was used for *in vitro* transcription in a 50 μl reaction containing 10 µl of T7 Transcription 5× Buffer, 16 µl of rNTPs (25 mM ATP, CTP, GTP, UTP), 19 µl of linear template DNA and 5 µl of Enzyme Mix (T7) at 37°C for 4 h, followed by treatment with 1 µl of RQ1 RNase-Free DNase at 37°C for 15 min to remove the DNA template. Approximately 15 µg of RNA transcript was transfected into Vero cells grown to 95% confluence in a 6-well plate using Lipofectamine^™^ 3000 reagent (Thermo Fisher Scientific), and then, the plate was incubated at 37°C for 48 h. The titer and phenotype of the recovered viruses were determined by plaque assay.

### Luciferase assay

293 T cells seeded in a 24-well plate were transfected with 1 µg of EV-A71-Fluc RNA using Lipofectamine^™^ 3000 reagent (Thermo Fisher Scientific) according to the manufacturer’s protocol. At 4 and 24 h post transfection, the cells were washed twice with PBS and lysed using cell lysis buffer (Promega). Luciferase signals were measured in a luminescence microplate reader (Monolight).

### Plaque assay and viral replication kinetics

The recovered viruses were inoculated with Vero cell monolayers at an MOI of 5 in triplicate wells in 12-well plates. After attachment at 37°C with 5% CO_2_ for 2 h, the inocula were removed, and the cell monolayers were washed twice with MEM. Then, 1 ml of MEM containing 2% FBS and 1% penicillin/streptomycin was added. The plates were incubated at 37°C with 5% CO_2_ for 72 h. At different time points during incubation, viruses were harvested from the cell culture medium. For the plaque assay, the viruses collected were serially diluted 10-fold eleven times in MEM containing 1% FBS and 1% penicillin/streptomycin. Two hundred microliters of diluted virus was added to each well of 12-well plates and swirled every 20 min to ensure complete coverage of the cell monolayers. After incubation at 37°C with 5% CO_2_ for 2 h and washing twice with MEM, the infected cell monolayers were overlaid with 1 ml of 1% low-melting-point agarose gels (Promega) containing 1× MEM (Gibco), 1% low-melting-point agarose, 1% FBS and 1% penicillin/streptomycin. The plates were incubated at 37°C with 5% CO_2_ for 48 h. Neutral red staining solution was added to each well, the visible plaques were counted, and viral titers were calculated.

### Immunofluorescence assay

For the immunofluorescence assay, transfected Vero cells were fixed in 100% methanol (chilled at −20°C) for 10 min. The cells were washed with PBS 3 times and blocked with blocking buffer containing 1% BSA at 37°C for 30 min. The cells were then incubated overnight at 4°C with mouse monoclonal antibody against EV-A71 VP1 (Abcam) diluted in PBS (pH 7.4) containing 1% bovine serum albumin (BSA) at 1:500. After washing with PBS, a goat anti-mouse IgG secondary antibody labeled with Alexa Fluor 488 (Thermo Fisher Scientific) was applied for 1 h at 37°C. The cells were then washed three times with PBS, and the nuclei were stained with 4’,6-diamidino-2-phenylindole (DAPI) (Abcam) following the manufacturer’s instructions.

### RNA-binding protein pulldown assay

The pGEM-3zf(+) plasmids containing the *cre* sequence or *cre* variants were extracted from bacterial strain Top10 and linearized by BamHI, followed by *in vitro* transcription. These *cre* RNAs were biotinylated by using a Pierce^™^ RNA 3’ End Biotinylation Kit (Thermo Fisher Scientific) following the manufacturer’s instructions. Biotinylated scramble RNA was used as a negative control (NC RNA). Proteins from T98G cells, Vero cells and KMB17 cells were separately extracted using 1 ml of IP lysis buffer (Thermo Fisher Scientific) supplemented with a protease inhibitor cocktail (Roche) and an RNase inhibitor (Thermo Fisher Scientific). To exclude the nonspecific binding of the extracted proteins and streptavidin agarose resin (Pierce) as much as possible, the protein extracts were first precleared by incubation with the agarose resin for 1 h at 4°C. Then, biotinylated *cre* RNAs, biotinylated NC RNAs or nonbiotinylated *cre* RNAs were separately added to 300 µl of the precleared supernatants. After incubation for 4 h at 4°C, streptavidin agarose resins were added. The mixtures were rotated for 1 h at 4°C followed by centrifugation. The pulldown products were washed three times with washing buffer and boiled before SDS-PAGE, and the gel was analyzed by Coomassie brilliant blue staining. Specific protein bands were excised, digested by trypsin, and identified by mass spectrometry.

### Western blot analysis

Cells were harvested and washed with PBS three times and then incubated with RIPA buffer (Thermo Fisher Scientific) supplemented with a protease inhibitor cocktail on ice for 30 min to extract cell proteins. The cell lysates were electrophoresed by 10% SDS-PAGE and transferred to PVDF membranes. The primary antibodies used for western blotting included rabbit monoclonal antibodies against IGF2BP2 (Abcam, 1:2,000 dilution) and ILF3 (ABclonal, 1:1,000), a rabbit polyclonal antibody against hnRNP H (Abcam, 1:2,000 dilution), and a mouse monoclonal antibody against β-actin (CWBIO, 1:5,000 dilution). The membrane was incubated with a primary antibody at 4°C overnight, followed by washing three times and an hour-long incubation with a secondary antibody conjugated with horseradish peroxidase. Blots were visualized by ECL development.

### RNAi

The siRNAs targeting IGF2BP2 and negative control siRNA (siNC) were designed by Thermo Fisher Scientific as follows: IGF2BP2 siRNA-1, 5’- GCCGUUGUCAACGUCACAU-3’; IGF2BP2 siRNA-2, 5’- CCCAGUUUGUUGGUGCCAU-3’; and siNC, 5’- ACGUGACACGUUCGGAGAAUU-3’. The siRNA was transfected into Vero cells in a 6-well plate using Lipofectamine^™^ 3000 reagent (Thermo Fisher Scientific) following the manufacturer’s instructions. At 24 h after transfection, the cells were collected for western blot detection.

### Reverse transcription and real-time PCR

KMB17 cells transfected with different siRNAs were infected with EV-A71 for 24 h. The culture media were collected for RNA extraction. cDNAs were prepared from 5 µl of RNA using 200 U of PrimeScript II RTase (Takara), 200 U of RNase inhibitor, dNTP mixture and oligo-dT primers at 42°C for 1 h. Real-time PCR was performed using SYBR Green Supermix (Applied Biosystems, US) according to the manufacturer’s protocol. The expression levels of EV-A71 in the supernatants of IGF2BP2-knockdown cells were normalized against NC cells.

### RNA structure prediction

RNAstructure software [33] was used to analyze the secondary structure of the EV-A71 genome and EV-A71-*cre* candidates. All settings were at its default values.

### Statistical analysis and graphs producing

The statistical analysis was performed by Microsoft Excel using a two-tailed Student’s t test. Values with p ≤ 0.05 were considered statistically significant. * *p* < 0.05, ** *p* < 0.01, *** *p* < 0.001. Illustrator software was used to further produce the graphs.

## Results

### A cre was identified within the 2C-coding regions of EV-A71

**Figure 1.**
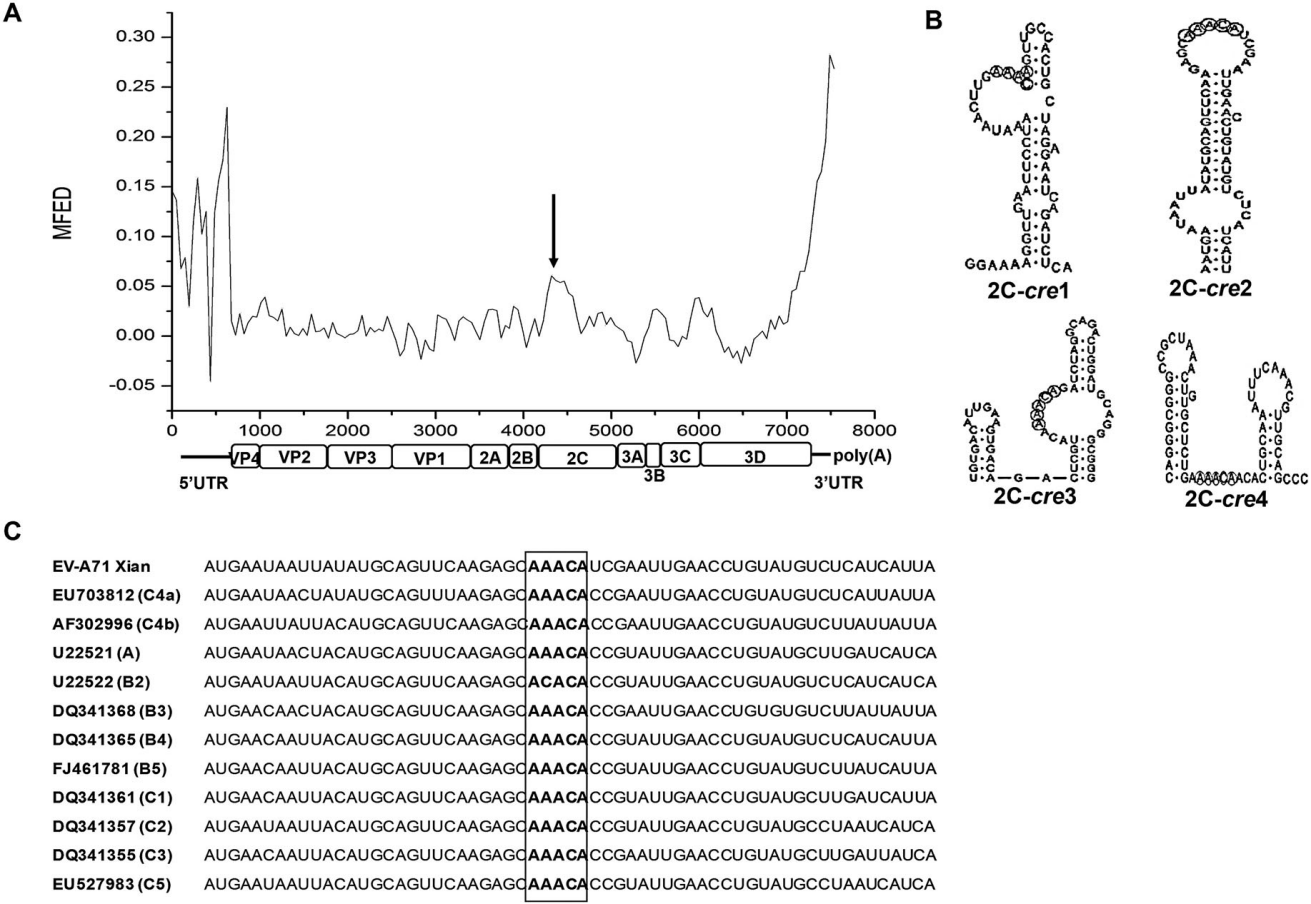
Recognition of the *cre* in the EV-A71 genome.

In picornaviruses, the *cre*s likely share a highly conserved secondary structure. To search for a potential *cre* in the EV-A71 genome, we used RNAstructure to analyze the secondary structure of the EV-A71 genome. The difference in minimum free energy (MFED) values indicated that the conserved stem-loop structure was within the EV-A71 RNA 2C-coding region (Figure 1A), which was then screened and shown to contain four positions with the core sequence AAACA (2C-*cre*1-4). Among these, 2C-*cre*2 (nt 4397-4457) with 61 nt in length was predicted to form a typical stem-loop structure, and its core sequence AAACA was in the loop which was consisted of 14 nt (Figure 1B). To determine the conservation of 2C-*cre*2, we aligned this region from different EV-A71 genotypes and found that 2C-*cre*2 with AAACA was conserved in all genotypes except genotype B2, in which the core sequence was ACACA (Figure 1C). Therefore, a *cre* is potentially located within the 2C-coding region of EV-A71 [EV-A71-*cre*(2C)]. Structural conservation indicated that this is a functionally important region of the EV-A71 genome and deserves further study.

A: Secondary structure analysis of the EV-A71 genome. The horizontal axis represented the EV-A71 genome and the locations of its protein-coding genes. The vertical axis represented MFED values. The higher MFED value was, the more likely the sequence was to form a secondary structure. B: Predicted secondary structures of the *cre* candidates, of which 2C-*cre*2 could form a typical stem-loop structure. C: 2C-*cre*2 with AAACA conserved among the genome sequences of different EV-A71 genotypes. The genotypes were indicated in the parentheses alongside the genbank accession numbers.

### The structure and core sequence of the cre affected the EV-A71 replication ability based on the EV-A71-Fluc replicon

**Figure 2.**
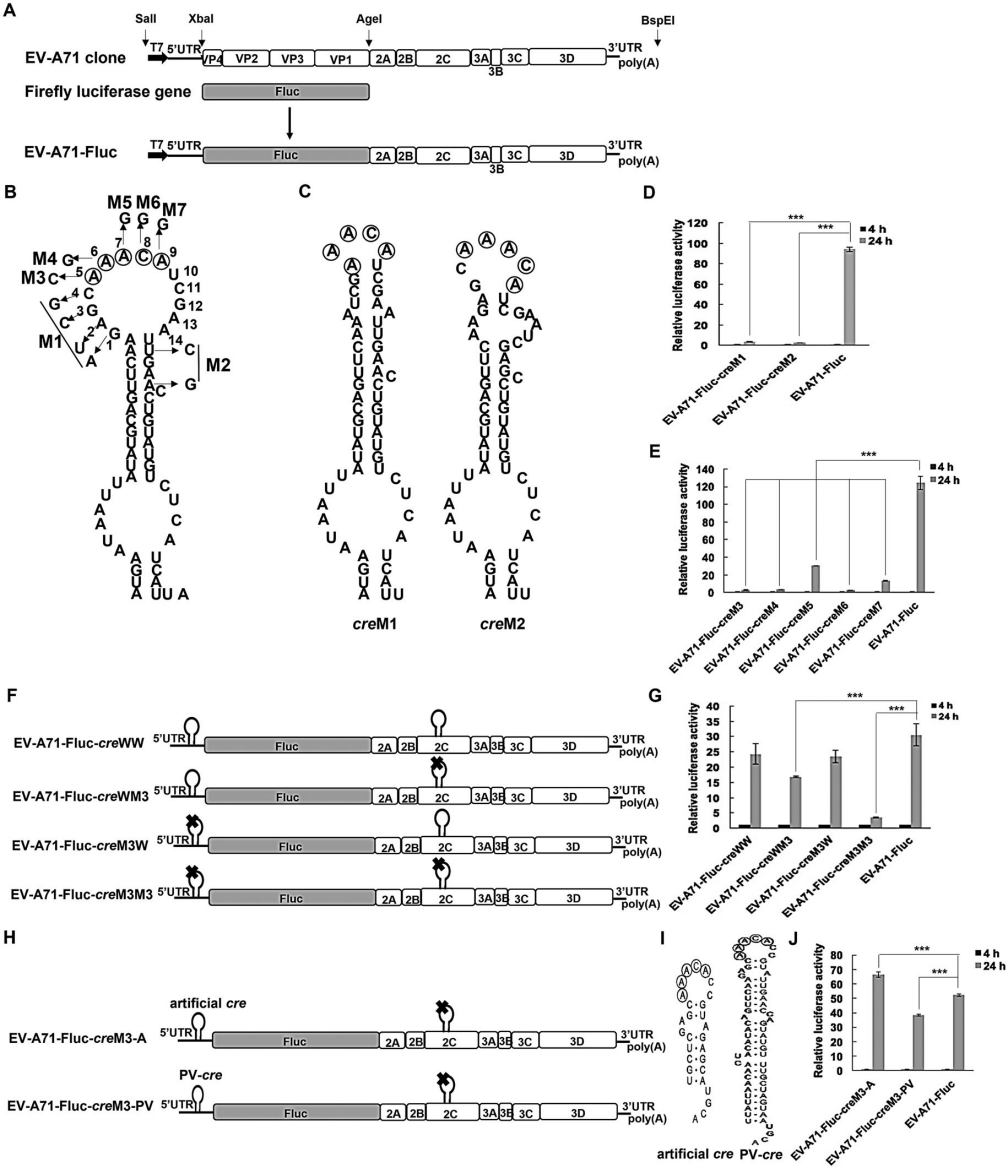
Effects of the *cre* on EV-A71 replication based on EV-A71 replicons.

Replicons have been used to easily and quickly study the *cis*-acting elements of viruses. Based on the full-length EV-A71 cDNA clone, we constructed an EV-A71 replicon (EV-A71-Fluc) with an internal in-frame deletion of the structural protein-coding regions, which were then replaced by a firefly luciferase reporter gene (Figure 2A). Because of the importance of the *cre* in virus replication, we wanted to determine whether the stem-loop structure and the core sequence AAACA were required for *cre* function. A total of seven mutations (M1-M7) were introduced into 2C-*cre*2 in the EV-A71 replicon (Figure 2B, C). Compared with that in cells with EV-A71-Fluc, luciferase activity was abolished at 24 h post transfection in 293 T cells with EV-A71-Fluc-*cre* M1 (_4420_GAGC_4423_ → _4420_AUCG_4423_) or M2 (U4435C, A4438G) (Figure 2D), which altered the secondary structure without amino acid changes in 2C, indicating that the function of EV-A71-*cre* was independent of the cognate amino acid sequence of the viral 2C protein. The effects of the mutations EV-A71-Fluc-*cre*s M3 (A4424C, K → Q), M4 (A4425G, K → R), M5 (A4426G), M6 (C4427G, H → D) and M7 (A4428G, H → R) were similar, showing significant interference with luciferase activities (Figure 2E).

Next, we hypothesized that the low luciferase activity after transfection was due to inactivation of the *cre* signal. We introduced a second copy of wild-type EV-A71-*cre* in the 5’UTR of replication-competent replicon EV-A71-Fluc (EV-A71-Fluc-*cre*WW) and the replication-incompetent replicon EV-A71-Fluc-*cre*M3 (EV-A71-Fluc-*cre*WM3) (Figure 2F), respectively, and these two dual-*cre* replicons were both active. When the second copy in the 5’UTR had the same mutation as the 2C region, however, the luciferase activity of the dual-*cre* replicon (EV-A71-Fluc-*cre*M3M3) was severely inhibited (Figure 2G). When the wild-type EV-A71-*cre* was restored in the 2C region in this inactive dual-*cre* replicon (EV-A71-Fluc-*cre*M3W), the luciferase activity was recovered (Figure 2G). These results indicated that the wild-type EV-A71-*cre* could restore the replication level of an inactive replicon independent of its location.

We were also interested in whether a heterologous *cre* could rescue the replication of the EV-A71-Fluc-*cre*M3. We constructed dual-*cre* replicons by either inserting an artificial *cre* consisting of an authentic EV-A71-*cre* core sequence and an artificial stem with an unrelated nucleotide sequence (EV-A71-Fluc-*cre*M3-A) or inserting a PV-*cre* (nt 4443-4509) (EV-A71-Fluc-*cre*M3-PV) between the cloverleaf region and internal ribosome entry site of the 5’UTR of the EV-A71-Fluc-*cre*M3 replicon (Figure 2H, I). The artificial *cre* and PV-*cre* were as functional as EV-A71-*cre* for the replication of dual-*cre* replicons (Figure 2J). These data indicated that the EV-A71-*cre* core sequence was critically important for replication and that a second copy of a *cre* of different picornaviral origin could rescue the replication-incompetent phenotype.

A: Construction of the EV-A71 replicon. B: Predicted secondary structure of EV-A71-*cre* (nt 4397-4457) in 2C and the mutants (M1-M7) used in this study. The core sequence of the EV-A71-*cre* was shown in the box. C: Predicted secondary structure of EV-A71-*cre*M1 and M2. D, E: Luciferase activities at 4 and 24 h after transfection of 293 T cells with EV-A71-Fluc-*cre*M1-M7 replicons. F, G: EV-A71-Fluc-*cre* constructs with dual-*cre*s in the 5’UTR and 2C region of the EV-A71 genome and their luciferase activity detected at 4 and 24 h post transfection of 293 T cells. H-J: Construction of EV-A71-Fluc-*cre* with the artificial *cre*-like sequence or PV-*cre* and their secondary structure. Luciferase activity was detected at 4 and 24 h post transfection of 293 T cells, normalized in 24 h to the luciferase activity at 4 h. The results are the averages of three independent cultures of transfected cells. Error bars indicate the standard deviations. Values with p ≤ 0.05 were considered statistically significant. * *p* < 0.05, ** *p* < 0.01, *** *p* < 0.001.

### Mutations in the cre core sequence affected the EV-A71 replication ability based on full-length cDNA clones

**Figure 3.**
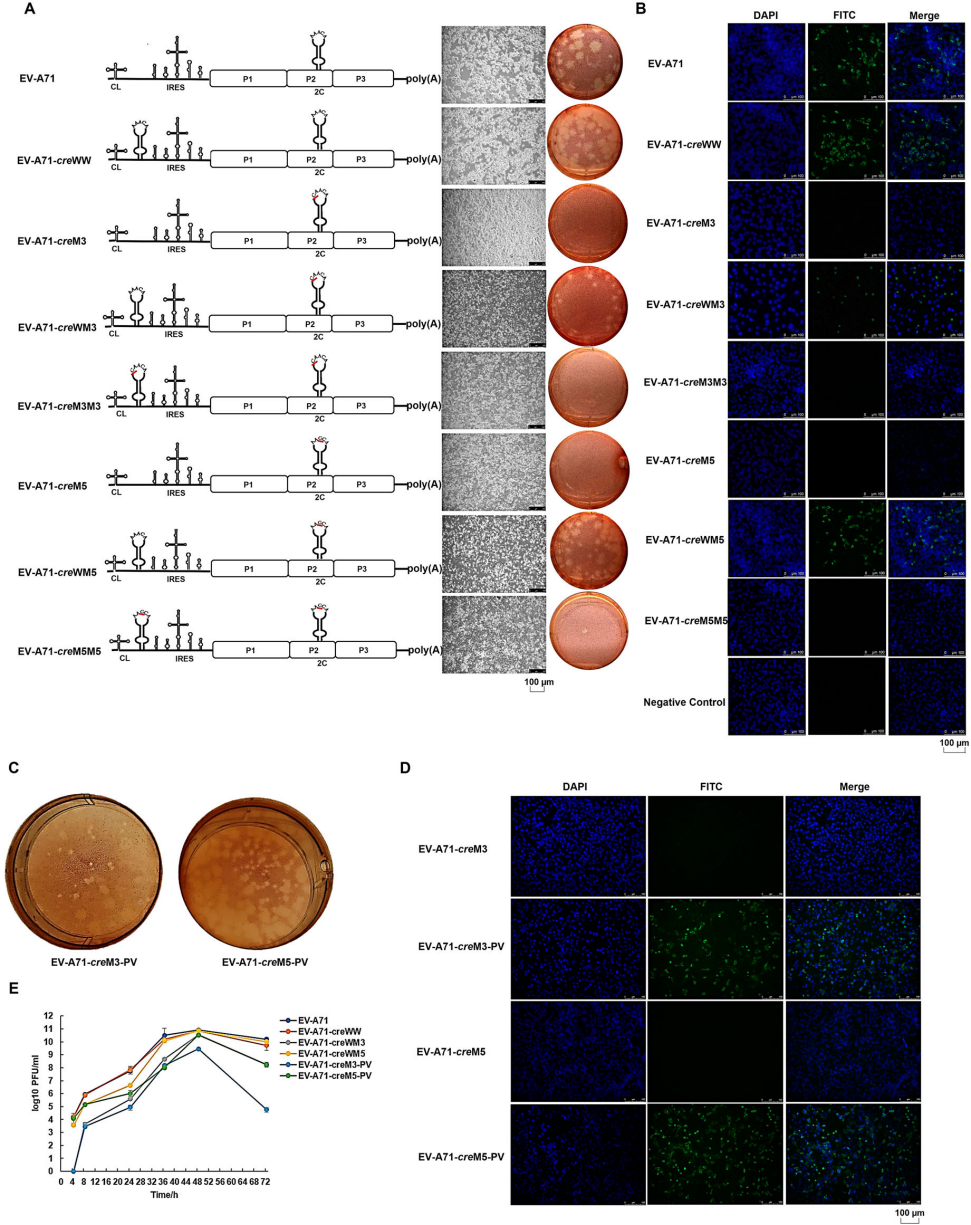
Effects of the *cre* on EV-A71 replication based on full-length cDNA constructs.

To better validate the effects of mutations within the core sequence of the EV-A71-*cre*, we constructed a series of full-length EV-A71 cDNA clones carrying single or dual *cre*s (Figure 3A). The mutations in the EV-A71-*cre*s WW, M3, WM3, M3M3 and M5 corresponded to the EV-A71-Fluc-*cre*s WW, M3, WM, M3M3 and M5 replicons, respectively. The EV-A71-*cre*M3 with the A4424C mutation (nonsynonymous mutation) and the EV-A71-*cre*M5 with the A4426G mutation (synonymous mutation) showed no cytopathic effects (CPEs) or immunofluorescence after 48 h of incubation with Vero cells at 37°C post transfection, suggesting that these mutant RNAs were impaired in replication. The dual-*cre* full-length EV-A71 cDNA clones with a second copy of the wild-type EV-A71-*cre* in the 5’UTR of the genome (EV-A71-*cre*s WW, WM3, and WM5) rather than the mutants (EV-A71-*cre*s M3M3 and M5M5) could be successfully rescued and developed CPEs (Figure 3A, B). Similar to previous findings with the dual-*cre* EV-A71 replicon, the PV-*cre* had a comparable ability of viral recovery (Figure 3C, D).

To exclude the possibility that a second *cre* insertion impaired replication of the rescued viruses, we also carried out one-step growth experiments to study the replication kinetics of these rescued viruses. Dual-*cre* viruses carrying wild-type EV-A71 *cre* in the 5’UTR without the A4424C mutation in the 2C region displayed growth kinetics similar to those of wild-type EV-A71. However, the titer of dual-*cre* viruses carrying the PV-*cre* in the 5’UTR (EV-A71-*cre*M3-PV and EV-A71-*cre*M5-PV) dropped rapidly after 48 h of infection, particularly when the viruses carried the A4424C mutation in the 2C region (*p* < 0.001) (Figure 3E). These results suggested that the A4424C mutation in the 2C region affected viral replication more severely than the A4426G mutation. Heterologous *cre*s, such as the PV-*cre*, might not be conducive to the maintenance of viral virulence.

A: A series of full-length EV-A71 cDNA constructs. The CPE and plaque results in Vero cells are shown on the right side. M3: A replaced by C at site 5 of the wild-type EV-A71-*cre*. M5: A replaced by G at site 7 of the wild-type EV-A71-*cre*. WW: the cDNA construct with two wild-type EV-A71-*cre*s separately in the 2C region and 5’UTR of the EV-A71 genome. WM: the cDNA constructs with the mutation of EV-A71-*cre* in the 2C region but with a second copy of the wild-type EV-A71-*cre* inserted in the 5’UTR of the EV-A71 genome. MM: the cDNA constructs with the mutations of EV-A71-*cre*s in the 2C region and 5’UTR. The red lines show the exact mutations. Scale bar: 100 µm. B: Infectivities of the full-length EV-A71 cDNA clones detected by an immunofluorescence assay (IFA) using an anti-EV-A71 VP1 monoclonal antibody. Scale bar: 100 µm. C, D: Rescued dual-*cre* viruses determined by plaque assay in Vero cells and by IFA using an anti-EV-A71 monoclonal antibody when the PV-*cre* was inserted into the 5’UTR of EV-A71 mutant clones. Scale bar: 100 µm. E: Replication kinetics of six rescued viruses. At certain time points (4, 8, 24, 36, 48 and 72 h) after infection with EV-A71-derived infectious clones, the cell culture media were collected, and the virus titer was determined in Vero cells (n = 3).

### Knockdown of the interaction of the cellular protein IGF2BP2 with the EV-A71-cre downregulated virus production

**Figure 4.**
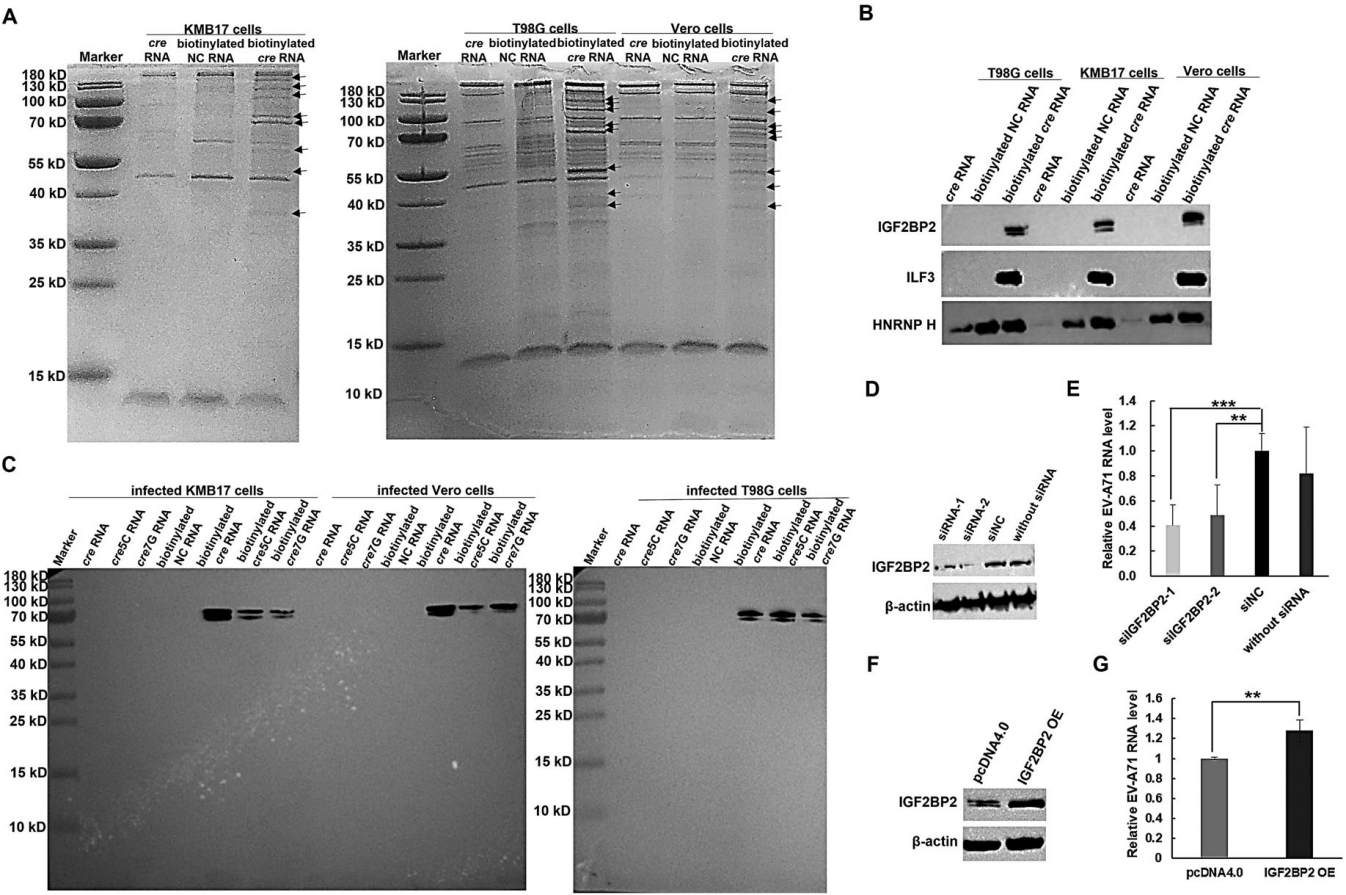
Interaction between the EV-A71-*cre* RNA and cellular proteins.

Although we observed significant effects of *cre* mutations on EV-A71 production, the possible specificity and conservation of the interaction between the EV-A71 *cre* and its cell hosts remained to be investigated. Biotinylated EV-A71-*cre* RNA probes were incubated with cellular proteins extracted from KMB17 cells, T98G cells and Vero cells. Nonbiotinylated *cre* RNA probes and biotinylated NC RNA probes were used as negative controls. Compared with the controls, the biotinylated EV-A71-*cre* RNA enriched 8 differential bands from each of these three types of cells, as observed by Coomassie brilliant blue staining (Figure 4A). By mass spectrometry, IGF2BP2, NF90/NF110 [encoded by the interleukin enhancer-binding factor 3 (*ILF3*) gene], heat shock proteins (HSPs), nucleolin, heterogeneous nuclear ribonucleoproteins (HNRNPs) and some other RNA-binding proteins (RBPs) were identified (Table S2). Due to the limited protein data in the database on *Cercopithecus aethiops* for analysis, we confirmed the conserved interactions mainly on the basis of proteins identified in human cells by western blot. The results showed that the binding of IGF2BP2 and ILF3 with the EV-A71-*cre* RNA was specific in all tested cells, while the binding of HNRNP H was nonspecific because of its binding with biotinylated NC RNA (Figure 4B).

To determine whether there was any relationship between the mutations in the core sequence AAACA of the EV-A71-*cre* and the binding of cellular proteins, *cre* RNAs with A4424C or A4426G mutation which impairing the replication of the resulting virus were used to assay bindings to IGF2BP2. Both mutations generally caused reduced binding to IGF2BP2 across samples from different infected cells (Figure 4C).

To investigate the effects of the IGF2BP2 protein on the production of EV-A71, we designed two siRNAs against human cellular IGF2BP2 and checked the level of EV-A71 after 24 h of infection of KMB17 cells. Compared with the negative control, the knockdown of IGF2BP2 expression in KMB17 cells resulted in a significant decrease in EV-A71 production (Figure 4D, E), while IGF2BP2 overexpression led to a significant increase in EV-A71 production (Figure 4F, G).

A: Cellular proteins extracted from T98G cells, Vero cells and KMB17 cells pulled down by a biotinylated EV-A71-*cre* RNA probe and observed by Coomassie brilliant blue staining. A nonbiotinylated *cre* RNA probe and biotinylated NC RNA probe were used as negative controls. Special protein bands indicated by arrows were excised and identified by mass spectrometry. B: The interactions of cellular proteins with the EV-A71 *cre* RNA probe were confirmed by western blot. C: Cellular proteins extracted from EV-A71-infected cells (T98G cells, KMB17 cells and Vero cells) pulled down by biotinylated EV-A71-*cre* RNA probes and detected by western blot using IGF2BP2 antibody. Nonbiotinylated *cre* RNA and biotinylated NC RNA were used as negative controls. 5C: A replaced by C at site 5 (nt 4424) of the wild-type *cre*, 7G: A replaced by G at site 7 (nt 4426) of the wild-type *cre*. D: Knockdown of IGF2BP2 expression levels in KMB17 cells by siRNAs checked by western blot. β-Actin was used as a loading control. E: Reduced EV-A71 RNA level after knockdown of IGF2BP2 expression in the culture media of KMB17 cells detected by real-time PCR (n=3). **: *p *< 0.01, ***: *p *< 0.001. F: Overexpression (OE) of IGF2BP2 expression level in KMB17 cells checked by western blot. The transfection of pcDNA4.0 plasmid was used as negative control. β-Actin was used as a loading control. G: Increased EV-A71 RNA level after IGF2BP2 overexpression in the culture media of KMB17 cells detected by real-time PCR (n=3). **: *p* < 0.01.

## Discussion

Over the past 20 years, the discovery of *cre* within various picornavirus genomes improves the understanding of picornavirus RNA replication. However, the location of the *cre* in the EV-A71 genome and its molecular mechanism are poorly understood. Here, we report a *cre* within the EV-A71 RNA 2C-coding region.

Evidence for the importance of the secondary structure and the AAACA sequence of *cre* involved in templating VPg uridylation has been gathered mainly in PV. We aligned *cre* sequences from different EV-A71 genotypes and found that the AAACA sequence located on the top of the hairpin, except for the ACACA sequence in genotype B2, likely corresponds to a common feature shared with the *cre*s of some other picornavirus, such as PV1, HRV2, FMDV and encephalomyocarditis virus (EMCV) [34], although the primary nucleotide sequences from different genera have substantial variations and the loops are variable in sequence and length. We identified that the EV-A71 *cre* is 61 nt in length and contains a top loop of 14 nt. Through a series of mutants derived from a replicon containing the firefly luciferase reporter gene in place of the EV-A71 P1 region, the silent mutations M1 and M2 altering the stem-loop structure of *cre* are likely to have profound effects on the replication efficiency. In addition, any of a single mutation of AAACA in the loop significantly inhibited the luciferase activity. These results led us to consider the importance of the stem structure and the bulging AAACA sequence of the EV-A71 cre for the replication phenotype. Given that only a 33-base segment is required to support HRV14 RNA replication [21], extensive studies may be needed to determine the minimal functional EV-A71-*cre*.

Reverse genetic systems provide a direct platform to explore the detailed molecular mechanisms responsible for EV-A71 pathogenesis and to develop countermeasures [35–39]. Consistent with the replicon mutants, we constructed EV-A71 full-length mutants with single *cre* or dual *cre*s. Both the A4424C and A4426G mutations abolished viral RNA infectivity. Although the A4424C mutation leads to a K → Q amino acid change in the ATPase domain of 2C, previous evidence indicates that this change does not significantly interfere with the synthesis or processing of the polyprotein or with the ATPase activity of 2C [29, 40]. The replication-incompetent effects caused by the A4424C mutation can be attributed to inactivation of the *cre* signal. The A4424C mutation in 2C has more severe effects than the A4426G mutation on viral replication, as indicated by the rapid decrease in the replication kinetics of the rescued dual-*cre* viruses carrying the A4424C mutation. We did not choose to change the second A of the AAA triplet for the construction of a full-length cDNA mutant since the loop in the presumptive *cre* in EV-A71 genotype B2 carries an ACACA instead of an AAACA sequence. A study on PV showed that mutation of either of the first two As of the AAA triplet within the loop of the PV-*cre* abolished PV infectivity, and the effect of mutating the third A of the AAA triplet was less severe [28]. These indicate that each of the exact nucleotide in the core sequence of picornaviral *cre*s may act with varying degree.

The variable location of the *cre* within picornaviral species suggests that its position within the RNA genome may not be particularly critical. Indeed, virus production with the EV-A71 genome carrying the lethal A4424C or A4426G mutation can be rescued by the insertion of either the wild-type *cre* from EV-A71 or a cognate *cre* from a PV in the 5’UTR. It indicates that the efficient rescuing function of the PV-*cre* is related to a functional *cre* with proper core sequence and secondary structure, and also in dependence on the interaction with the factors involved in VPg uridylation. The positional independenceof the *cre* has also been demonstrated in PV1, FMDV and hepatitis A virus (HAV) [23, 24, 27, 31]. It is intriguing to speculate that the dual *cre*s with the insertion of the Sabin vaccine strain *cre* in the 5’UTR of the EV-A71 genome may contribute to the attenuation phenotype of EV-A71.

The host cell machinery is required and rewired for viral replication [41–44]. Studies on host-virus interactions improve our understanding of the EV-A71 life cycle and are invaluable in identifying potential drug targets, which will very likely be relevant to other related picornaviruses. Our results reveal that the host factor IGF2BP2 specifically associates with the EV-A71-*cre* RNA. IGF2BP2 is an RNA-binding protein and lies upstream of both the mitogen-activated protein kinase (MAPK) and phosphatidylinositol 3-kinase (PI3 K) signaling pathways [45]. IGF2BP2 negatively regulates the release of preformed viruses by infected cells and restrains the production of infectious HCV particles [46]. An siRNA targeting IGF2BP2 appears to affect the early stage of the life cycle of influenza virus [47]. Here, knockdown of IGF2BP2 expression decreased EV-A71 production, and vice versa, suggesting a cooperative relationship between the EV-A71-*cre* RNA and IGF2BP2. Due to the ability of PV-*cre* to rescue the replication-incompetent phenotype, it is speculated that PV-*cre* may mediate the recovery through interaction with IGF2BP2. In addition to IGF2BP2, ILF3, HSPs and HNRNPs were shown to potentially associate with the EV-A71-*cre* RNA in our study. HNRNP C has been reported to functionally interact with the 5’ end of poliovirus negative-strand RNA and encourages efficient positive-strand RNA synthesis [42, 48]. Additional experiments looking into HNRNPs and the interaction between HNRNPs and IGF2BP2 in EV-A71 infection should be carried out. Their roles in viral infection and the detailed signaling pathway remain to be elucidated.

In summary, the EV-A71-*cre* within the 2C-coding region of the genome shows both significant similarities and differences in comparison to the related features of other picornaviral genera. The EV-A71-*cre* is location independent but functionally important. The study of this essential replication element and its interaction with the cell host enhances our understanding of the molecular virology of EV-A71 and provides knowledge regarding picornaviral *cre*s.

## Supplementary Material

Supplemental_Information.doc
