# Supplementary material for: A single mutation in the cis-acting replication element identified within the EV-A71 2C-coding region causes defects in virus production in cell culture
Source: Emerg Microbes Infect. 2021 Oct 17;10(1):1988–99. doi: 10.1080/22221751.2021.1977590 (PMC8526025; doi:10.1080/22221751.2021.1977590)
Supplement: Supplemental_Information.doc [file TEMI_A_1977590_SM2597.doc]

**Supplemental Information**

**Table S1. Primers used for the construction of EV-A71 *cre* mutants**

| **Primer name** | **Primer sequence (5’ → 3’)** |
| --- | --- |
| EV-A71 5’ UTR-XbaI For | AGTCTGTGACCACCCTTATCTAGATCTTGACCCTTAACAC |
| EV-A71 5’ UTR-XbaI Rev | GTGTTAAGGGTCAAGATCTAGATAAGGGTGGTCACAGACT |
| EV-A71-*cre*M1 For | GAAGAGAATGAATAATTATATGCAGTTCAAATCGAAAC**ATCG**AATTGAACCTGTATGTCTCATC |
| EV-A71-*cre*M1 Rev | GATGAGACATACAGGTTCAATT**CGAT**GTTTCGATTTGAACTGCATATAATTATTCATTCTCTTC |
| EV-A71-*cre*M2 For | ATATGCAGTTCAAGAGCAAACATCGAAT**C**GA**G**CCTGTATGTCTCATC |
| EV-A71-*cre*M2 Rev | GATGAGACATACAGG**C**TC**G**ATTCGATGTTTGCTCTTGAACTGCATAT |
| EV-A71-*cre*M3 For | ATAATTATATGCAGTTCAAGAGC**C**AACATCGAATTGAACCTGTATGT |
| EV-A71-*cre*M3 Rev | ACATACAGGTTCAATTCGATGTT**G**GCTCTTGAACTGCATATAATTAT |
| EV-A71*-cre*M4 For | ATAATTATATGCAGTTCAA**G**AGCAGACATCGAATTGAACCTGTATGT |
| EV-A71-*cre*M4 Rev | ACATACAGGTTCAATTCGATGTCTGCT**C**TTGAACTGCATATAATTAT |
| EV-A71-*cre*M5 For | ATTATATGCAGTTCAAGAGCAA**G**CATCGAATTGAACCTGTATGTC |
| EV-A71-*cre*M5 Rev | GACATACAGGTTCAATTCGATG**C**TTGCTCTTGAACTGCATATAAT |
| EV-A71-*cre*M6 For | ataattatatgcagttcaagagcaaa**g**atcgaattgaacctgtatgt |
| EV-A71-*cre*M6 Rev | acatacaggttcaattcgat**c**tttgctcttgaactgcatataattat |
| EV-A71-*cre*M7 For | tatatgcagttcaagagcaaac**g**tcgaattgaacctgtatgtc |
| EV-A71-*cre*M7 Rev | gacatacaggttcaattcga**c**gtttgctcttgaactgcatata |
| EV-A71-*cre*-NsiI For | **T**ATGAATAATTATATGCAGTTCAAGAGCAAACATCGAATTGAACCTGTATGTCTCATCATTA**ATGCA** |
| EV-A71-*cre*-NsiI Rev | **T**TAATGATGAGACATACAGGTTCAATTCGATGTTTGCTCTTGAACTGCATATAATTATTCAT**ATGCA** |
| EV-A71-*cre*PV-NsiI For | **T**TATTAACAACTACATACAGTTCAAGAGCAAACACCGTATTGAACCAGTATGTTTGCTAGTA**ATGCA** |
| EV-A71-*cre*PV-NsiI Rev | **T**TACTAGCAAACATACTGGTTCAATACGGTGTTTGCTCTTGAACTGTATGTAGTTGTTAATA**ATGCA** |
| EV-A71-*cre*Art-NsiI For | **T**GCTCGAGCAAACACCGTAGAGC**ATGCA** |
| EV-A71-*cre*Art-NsiI Rev | **T**GCTCTACGGTGTTTGCTCGAGC**ATGCA** |
| EV-A71-Fluc-XbaI For | **TCTAGA**ATGGAAGACGCCAAAAACATAAAG |
| EV-A71-Fluc-AgeI Rev | **ACCGGT**CACGGCGATCTTTCCGCCCTTC |

**Table S2. Potential EV-A71 *cre* RNA binding proteins identified by MS**

| **Protein ID** | **ProteinName** | **Gene ID** | **Gene Name** |
| --- | --- | --- | --- |
| Q00839 | Heterogeneous nuclear ribonucleoprotein U | 3192 | HNRNPU |
| F4ZW66 | NF110b |  |  |
| F4ZW65 | NF90b |  |  |
| B3KTP9 | cDNA FLJ38578 fis, clone HCHON2007674, highly similar to NUCLEOLIN |  |  |
| O60506 | Heterogeneous nuclear ribonucleoprotein Q | 10492 | SYNCRIP |
| Q9Y6M1 | Insulin-like growth factor 2 mRNA-binding protein 2 | 10644 | IGF2BP2 |
| O00425 | Insulin-like growth factor 2 mRNA-binding protein 3 | 10643 | IGF2BP3 |
| Q9NZI8 | Insulin-like growth factor 2 mRNA-binding protein 1 | 10642 | IGF2BP1 |
| A0A0J9YXX5 | Poly(U)-binding-splicing factor PUF60 |  | PUF60 |
| D6RBM0 | Heterogeneous nuclear ribonucleoprotein H |  | HNRNPH1 |
| Q53FG3 | Interleukin enhancer binding factor 2 variant | 3608 |  |
| F8W1T6 | RNA-binding motif, single-stranded-interacting protein 2 |  | RBMS2 |
| B2R7W4 | cDNA, FLJ93632, highly similar to Homo sapiens heterogeneous nuclear ribonucleoprotein R (HNRPR), mRNA |  |  |
| A0A499FI31 | Squamous cell carcinoma antigen recognized by T-cells 3 | 9733 | SART3 |
| P08238 | Heat shock protein HSP 90-beta | 3326 | HSP90AB1 |
| Q9BSV4 | SFPQ protein |  | SFPQ |
| Q9Y3F4 | Serine-threonine kinase receptor-associated protein | 11171 | STRAP |
| O60506 | Heterogeneous nuclear ribonucleoprotein Q | 10492 | SYNCRIP |
| A0A024RDB4 | Heterogeneous nuclear ribonucleoprotein D (AU-rich element RNA binding protein 1, 37kDa), isoform CRA_c | 3184 | HNRPD |
| P09651 | Heterogeneous nuclear ribonucleoprotein A1 | 3178 | HNRNPA1 |
| P22626 | Heterogeneous nuclear ribonucleoproteins A2/B1 | 3181 | HNRNPA2B1 |
| Q9UKM9 | RNA-binding protein Raly | 22913 | RALY |
| H6U5Q1 | Nucleolin |  |  |
